# Efficacy in Using Urodynamic Parameters of Intravesical Electrical Stimulation for Detrusor Underactivity

**DOI:** 10.5152/tud.2024.23235

**Published:** 2024-03-01

**Authors:** Rahmat Aidil Fajar Siregar, Hendy Mirza, Widyawan Hami Seno, Nugroho Purnomo, Moammar Andar Roemare Siregar, Andika Afriansyah

**Affiliations:** 1 Division of Urology, Department of Surgery, Persahabatan General Hospital,Jakarta, Indonesia; 2 Division of Urology, Department of Surgery, Persahabatan General Hospital - Faculty of Medicine, Universitas Indonesia, Jakarta, Indonesia

**Keywords:** Intravesical electrical stimulation, detrusor underactivity, prospective study

## Abstract

**Objective:**

Intravesical electrical stimulation (IVES) remains a controversial therapy for detrusor underactivity (DUA). The purpose of this study is to determine the efficacy of IVES in patients with DUA using pre- and post-IVES urodynamic parameters.

**Methods:**

Intravesical electrical stimulation procedure is performed using a specific catheter equipped with an internal electrical electrode (cathode). The anode is subsequently affixed to the lower abdomen (suprapubic). Afterward, this specialized catheter is connected to a stimulator. Patients undergo a series of 12 IVES procedures in 1 month with the following predetermined parameters: 20 mA amplitude, 20 Hz frequency, 200 µs pulse width, and 60 minutes stimulation time. Patients underwent a follow-up urodynamic examination 1 month after the IVES series is completed.

**Results:**

After IVES, several notable changes were observed, including an increase in Qmax from 7.28 ± 5.24 to 7.29 ± 4.09 (*P* = .030), a decrease in post-void residual (PVR) from 73.03 ± 43.91 to 62.07 ± 39.10 (*P* = .005), and an increase in PDet@tQmax from 17.10 ± 12.35 to 18.87 ± 12.47 (*P* = .009). The aetiologies of DUA were categorized into 3 groups: chronic obstruction (CO), idiopathic (Idio), and neurological disorder (ND). The CO group exhibited significant changes in urodynamic parameters, specifically Qmax (*P* = .001), PVR (*P* = .001), and PDet@Qmax (*P* = .035). Similarly, the idiopathic group also demonstrated improvements in Qmax (*P* = .008), PVR (*P* = .037), and PDet@Qmax (*P* = .033).

**Conclusion:**

Intravesical electrical stimulation has been shown to have a positive effect on patients diagnosed with DUA, particularly those whose DUA is idiopathic or due to chronic obstruction.

**main points**

- IVES improves urine flow and detrusor pressure while reducing post-void residual in DUA patients.
- Efficacy of IVES varies significantly, showing improvements in cases of DUA due to chronic obstruction and idiopathic reasons but not in neurological disorders.
- No adverse effects were reported, indicating IVES as a safe and well-tolerated therapy option for patients.
- Results show significant improvements in urodynamic parameters, affirming the potential of IVES in DUA management.

## Introduction

Detrusor underactivity (DUA), as defined by the International Continence Society (ICS), is characterized by a decrease in the strength or duration of contractions, resulting in a failure or delayed bladder emptying duration compared to the typical voiding time.^1^ The precise etiology of DUA remains ambiguous. The diminished contractile capacity of the bladder, leading to voiding difficulties or disruptions in typical voiding patterns, may be attributed to alterations in its structural or functional anatomy.^2^ Currently, there are multiple documented factors contributing to DUA, including persistent bladder outlet obstruction (BOO), diabetes mellitus (DM), and neurological diseases (such as Parkinson’s disease, multiple sclerosis, herpes zoster, spinal cord injury, or peripheral nerve disorders). The International Continence Society, however, does not categorize DUA based on its underlying cause.^3^

Patients suffering from DUA often report subjective problems, such as feeling as though they have not completely emptied their bladder or that there is residual urine left after urination. Additionally, patients may describe the need for increased effort to successfully complete the voiding process.^4^ A DUA diagnosis is confirmed by conducting a urodynamic examination, which provides insights into the decreased contractions of the detrusor muscle, resulting in a decreased urine flow and detrusor pressure. Consequently, this condition hinders the ability to fully empty the bladder within the expected duration.^5^

Treatment modalities for DUA can be classified into various approaches, encompassing conservative strategies and clean intermittent catheterization, as well as pharmacotherapy involving the administration of medications such as alpha-blockers, cholinesterase inhibitors, muscarinic agonists, prostaglandin E2, and acotiamide. Surgical interventions include sacral nerve stimulation via electrical stimulation, injections into the external sphincter, procedures to address BOO, reduction cystoplasty, and latissimus dorsi detrusor myoplasty. Further, scientific investigations are currently being conducted to explore the potential of stem cell and gene treatments in the management of DUA.^6^ The European Urology Association Guidelines recommend intravesical electrostimulation (IVES); however, it only receives a weak strength rating.^7^

Saxtorph invented IVES, an active electrocatheter that treats urinary retention by implanting a neutral electrode under the skin and an active electrocatheter into the bladder.^8^ Intraluminal electrotherapy was first described by Katona and Berenyi in 1959 as a treatment for a number of gastrointestinal illnesses. Later, Katona used this method widely for neurogenic bladder dysfunction in 1975. Following that, the use of intraluminal electrotherapy to treat voiding disorders has grown.^9^

Patients with DUA have reportedly experienced positive effects after IVES. Improvements in urinary flow, post-voiding residual, voiding efficiency, and first sensation volume during the bladder filling phase were observed in the evaluation of urodynamic parameters following the IVES procedure.^10,11^ In this study, we evaluate the efficacy of IVES by comparing urodynamic parameters before and after the IVES procedure. In previous studies, efficacy assessment was done by observing clinical changes in clean intermittent catheterization (CIC) status and subjective assessments of quality of life. With the comparison of urodynamic parameters, the efficacy of IVES can be supported more objectively. This study aims to assess the efficacy of IVES in patients with DUA by comparing urodynamic parameters before and after the procedure.

## Material and Methods

### Study Design

This study is a single-center prospective cohort study conducted from May 23, 2021, to May 23, 2023, at Persahabatan Hospital in Jakarta. Approval from the ethics committee Persahabatan Hospital under ethical clearance number UM/1.20/000079/2023 was obtained at all study sites before the initiation of the study, and all participants provided written informed consent prior to the beginning of the study (Approval No: 79; Date: May 11, 2021). The study was conducted in accordance with the Declaration of Helsinki and Good Clinical Practice guidelines. Subjects were recruited using a consecutive sampling technique with a minimum sample size of 51 using a comparing 2 means formula with a confidence interval of 95% and a power of the sample size of 80%.

## Device Introduction

**Figure 1.**
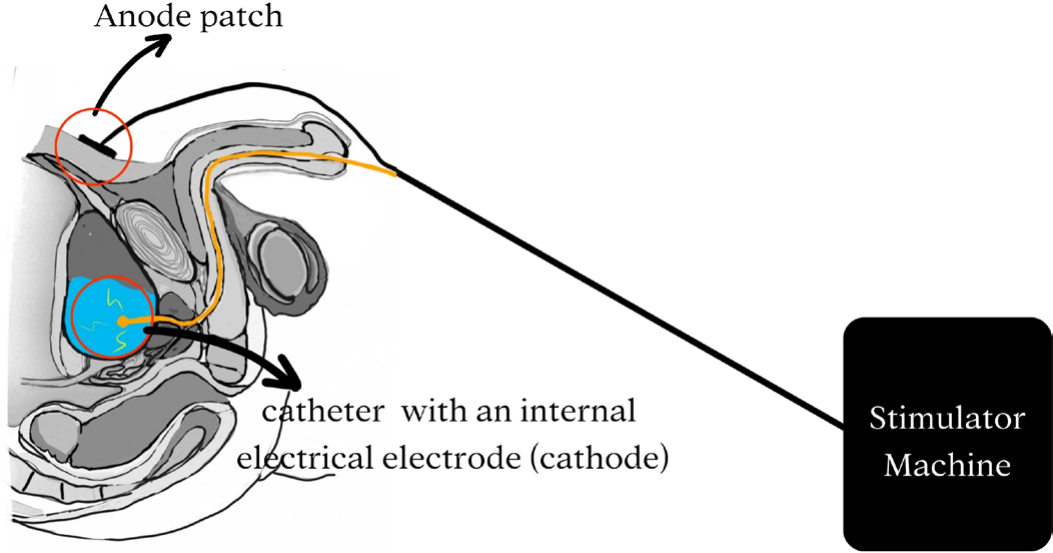
A schematic figure for intravesical electrostimulation (IVES) procedure. The IVES procedure involves inserting a sterilized, active electrocatheter through the urethra into the bladder.

The IVES procedure is performed using an IVES-specific catheter manufactured by Persahabatan Hospital in 5020 Salzburg, Austria, under the name Persahabatan Hospital. This specific catheter is equipped with an internal electrical electrode (cathode). The anode is subsequently placed on the lower abdomen (suprapubic) using gauze soaked in 0.9% NaCl. This catheter was then connected to a stimulator machine (Persahabatan Hospital) (Figure 1).

### Inclusion and Exclusion Criteria

The inclusion criteria are:

1. Patients with DUA diagnosed with urodynamic study. In males, detrusor underactivity was defined as bladder contractility index of less than 100; and in females as Qmax of 12 mL/s or less, and PDet@Qmax of 10 cmH_2_O or less.^12^
2. Detrusor underactivity with all etiologies.
3. Patients still have a sensation during bladder filling.
4. Intact innervation in the S2-S4 dermatomes, as indicated by the presence of contact sensation.

The exclusion criteria are:

1. Detrusor underactivity with atonic bladder (no detrusor contraction).
2. Cognitive impairment.
3. Detrusor underactivity combined with BOO (benign prostatic hypertrophy, bladder neck contracture, urethral stenosis, or prostatic cancer).
4. Detrusor underactivity accompanied by stones, tumors, or urinary tract infections.
5. Patients with severe cerebrovascular or cardiac disease, renal dysfunction, and pregnancy.

Subjects were classified into 3 primary groups according to the etiology of their DUAs: those who had prolonged BOO after treatment were categorized into the chronic obstruction (CO) group; those with DUA who had a history of normal delivery, cesarean delivery, or whose DUA is due to unknown etiology were classified into the idiopathic (Idio) group; and the neurological disorder (ND) group consisted of those diagnosed with canal stenosis, post-cerebrovascular accidents, and polyneuropathy resulting from chronic DM.

### Intravesical Electrostimulation Technique and Parameter Setting

Every subject who meets the criteria underwent 12 daily IVES procedures in 1 month with the following predetermined frequency parameters: 20 mA amplitude, 20 Hz frequency, 200 µs pulse width, and a 60-minute duration for each session. The procedure is stopped if a patient experiences unbearable heat, burning sensations, or discomfort. The follow-up urodynamic examination is performed 1 month after IVES.

### Urodynamic Study and Statistical Analysis

Subjects underwent urodynamic study (UDS) as a diagnostic evaluation for DUA. The urodynamic study adhered to the ICS guidelines for Good Urodynamic Practice to ensure quality control and proper reporting. In male patients, the diagnosis of DUA was established if the bladder contractility index was below 100. In female patients, DUA was defined as PDet@Qmax of ≤10 cmH_2_O and Qmax of ≤12 mL/s. Patients underwent the UDS evaluation 1 month after the final session of IVES. The efficacy of IVES was assessed based on the pre- and post-IVES UDS parameters.

Collected data are presented as mean and standard deviation. Statistical analysis on the pre- and post-IVES urodynamic parameter data were conducted using the paired sample *t*-test. A significance level of less than .05 (*P* < .05) was considered statistically significant.

## Results

A total of 58 subjects met the provided inclusion criteria. Data were collected through urodynamic assessments, a review of medical records, and physical examinations.

The mean age of the 58 subjects who met the inclusion criteria was 54.55 years old (Table 1). Thirty-three subjects (56.9%) were male, representing the majority of the subjects, and the remaining 25 (43.1%) were female. For 28 (48.3%) subjects, post-treatment prolonged BOO was the primary cause of their DUA.

Of the 58 subjects, only 2 did not use CIC, while 56 subjects relied on CIC to empty their bladders (Table 2). Thirty-five subjects out of 56 were utterly dependent on CIC. After the IVES procedure, 1 subject no longer required CIC, while 11 subjects who were utterly dependent became partly dependent on CIC, and the rest remained utterly dependent. Of the 21 subjects who were partly dependent on CIC, 6 became completely independent of CIC after treatment with IVES.

Table 3 presents a comparison of urodynamic parameters before and after the IVES procedure. Significant results include an elevation in Qmax (an increase from 7.28 ± 5.24 to 7.29 ± 4.09, *P* = .030) after IVES. There was a significant reduction in PVR from 73.03 ± 43.91 to 62.07 ± 39.10 (*P* = .005). The PDet@Qmax also has significant changes from 17.10 ± 12.35 to 18.87 ± 12.47 (*P* = .009). There were no statistically significant changes seen in other measures, including voided volume, first sensation, strong sensation, and cystometric capacity.

For the CO group, the Qmax (*P* = .001) and PDet@Qmax (*P* = .035) parameters show improvement post-IVES, as indicated by a higher median flow rate. The PVR measurements for this group show a decrease after IVES (*P* = .001). These results imply that IVES may enhance the bladder’s ability to expel urine in patients in the CO group.

Similarly, the Idio group, comprising patients with idiopathic etiology, also shows notable changes post-IVES. The Qmax (*P* = .008), PVR (*P* = .009), and PDet@Qmax (*P* = .033) all show a significant value change.

Differing from the CO and Idio groups, the ND group exhibited no statistically significant changes after undergoing IVES. In the ND group, the Qmax (*P* = .134), PVR (*P* = .239), and PDet@Qmax (*P* = .981) all did not show any significant changes, similar to other urodynamic parameters.

## Discussion

Post-IVES, there were changes in clinical parameters such as a decrease in the number of CIC uses and urodynamic parameter changes, particularly in Qmax, PVR, and PDet@Qmax post-IVES. eIntravesical lectrostimulation has a positive effect on enhancing bladder function in the CO and Idio group, compared to the ND group, which did not show any significant changes to indicate improvement in bladder function.

The micturition process starts with the stretching of the urinary bladder, which initiates afferent stimuli to begin the micturition reflex. This results in the relaxation of the internal sphincter and the subsequent influx of urine into the bladder neck. The presence of a distended bladder neck initiates the micturition reflex, leading to the contraction of the detrusor muscle.^13,14^ Intravesical electrostimulation works through a process that provides repetitive stimulation to the Aδ mechanoreceptor fibers.^15^ This process serves to amplify the intensity or precision of the signals, resulting in heightened responsiveness of the central micturition reflex pathway.^16^

**Figure 2.**
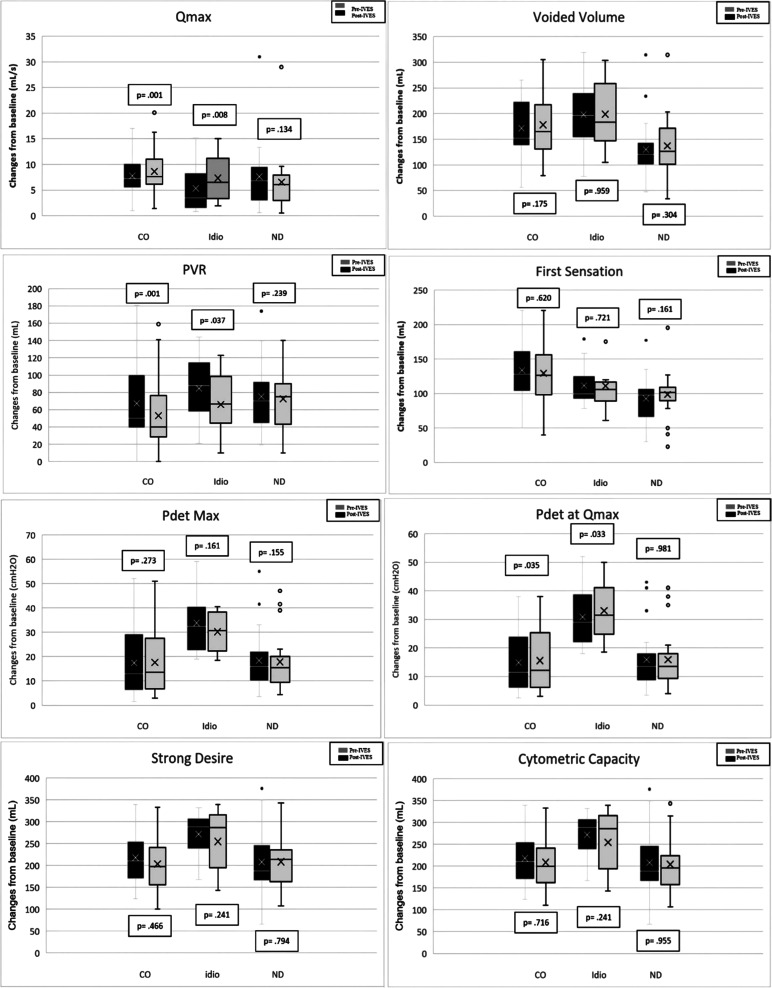
Boxplot of each urodynamic parameter. CO, chronic obstruction; Idio, idiopathic; ND, neurological disorder; PVR, post-void residual; Qmax: maximum flow rate; voided volume, the total volume of urine expelled; first sensation, the initial sensation of bladder filling; strong sensation, the volume at which a person feels a strong desire to void during cystometry; cystometric capacity, the maximum bladder capacity; PDet@Qmax, detrusor pressure at the maximum flow rate; PDetMax, maximum detrusor pressure. Black box: before IVES; Gray box: after IVES. *Wilcoxon signed-rank test; *P* < .05.

Patients who have been diagnosed with diabetic neuropathy may encounter impairments in the functioning of tiny, little myelinated Aδ fibers and unmyelinated C fibers.^17^ Bladder hyporeflexia may manifest in individuals with a prior history of stroke, possibly resulting from the presence of concomitant neuropathy or the use of certain medications.^18^ In the context of canal stenosis, individuals may have problems related to the parasympathetic efferent nerves originating from the sacral cord at the levels of S2-S4, as well as the sympathetic efferent nerves originating from the intermediolateral gray column at the levels of T11-L2.^19^ As a consequence of the neurological abnormalities, it is evident that the IVES stimulation procedure is incapable of enhancing the reactivity of the central micturition reflex pathway. Consequently, there are no notable changes noticed in the ND group when comparing the pre- and post-IVES urodynamic parameters. Based on the findings of the study, the boxplot shown in Figure 2 reveals that there are no statistically significant changes seen across all parameters within the ND group. Patients in this group also subjectively did not feel any change after undergoing the IVES procedure.

Compared to the findings of this study, which demonstrated no statistically significant changes in the ND group, previous studies conducted by Liao et al^10^ and Deng et al^11^ showed positive effects in patients who might have problems in impulse transmission that involve the micturition reflex pathway, such as those with spinal cord injury, spinal canal stenosis, and postoperative lumbar disc herniation.

In contrast to the ND group, both the CO and Idio groups showed an increase in Qmax and PDet@Qmax, together with a decrease in PVR. These findings suggest a positive impact in enhancing bladder function. Patients diagnosed with CO have histological structural abnormalities in the bladder; however, it is important to note that these alterations do not result in any significant modifications or disruptions to the neurological structures.^20^ In patients with CO, a decompensation event occurs, particularly the reduced sensitivity of afferent innervation due to thickening of epithelial cells, leading to disturbances in the contractions of smooth muscle in the bladder. This disruption affects the voiding phase during micturition.^21^ Intravesical electrostimulation works to enhance the sensitivity of these afferents, thereby improving the contraction process during bladder emptying.^22^

Patients whose DUA is of unknown etiology are categorized into the Idio group in this study. The potential cause of DUA in this group may be structural changes, including decreased axon density, resulting in impaired autonomic bladder innervation. These changes can cause diminished sensory function, contributing to the development of DUA.^23^ Intravesical electrostimulation works by providing repetitive stimulation to the Aδ mechanoreceptor fibers, addressing the issue of reduced bladder sensation in the idiopathic group, ultimately leading to an improvement in nerve function.^15^

Previous studies have established that IVES have positive effects. Other studies have shown positive benefits subsequent to a 3-week course of treatment.^24^ Several studies have also shown the positive benefits of a series of 10-15 sessions of IVES.^25^ This study was conducted for a duration of 1 month, during which the participants had a total of 12 IVES procedures on a daily basis. During the treatment period, no patients were observed to exhibit allergic responses, including symptoms such as itching, heat, or any other local or systemic manifestations.

The strength of this study was that it compares urodynamic parameters pre- and post-IVES to observe the efficacy of IVES, which has not been reported in previous research. However, the limitation of this study was that very strict inclusion criteria were applied for patients undergoing IVES. Detrusor underactivity actually encompasses a wide range of diagnostic criteria, from types of decreased contractility to bladder atonia. The efficacy depicted in this study only reflects the inclusion criteria of this research and does not represent the whole DUA population. Since this paper is only an observational study, we recommend that future research should include clinical trials to allow for comparisons between each group and a control or placebo.

In summary, IVES has been shown to have a positive effect on patients diagnosed with DUA, particularly those whose DUA is idiopathic or occurs due to chronic obstructions. However, the procedure does not seem to provide the same positive outcomes for patients whose DUA is due to neurological disorder. None of the patients was noted to exhibit any adverse effects while undergoing the IVES procedure.

## Figures and Tables

**Table 1.** Patient Demographics

Variables	Number of Subjects N = 58
Gender	
Male, n (%)	33 (56.9%)
Female, n (%)	25 (43.1%)
Age, years	54.55 (19.7)
Etiology of DUA, n (%)	
Prolonged bladder outlet obstruction after treatment	28 (48.3%)
Canal stenosis	10 (17.3%)
Post-cerebrovascular accidents	6 (10.3%)
Polyneuropathy due to prolonged diabetes mellitus	4 (6.9%)
Post-normal labor	4 (6.9%)
Post-cesarean section	2 (3.4%)
Idiopathic	4 (6.9%)

DUA, detrusor underactivity.

**Table 2.** Changes in Pre- and Post-Intravesical Electrostimulation Clean Intermittent Catheterization Status

CIC dependency category	Pre-IVES Sample Count	Post-IVES	Post-IVES Subtotal
Independent	2	–	2
Utterly dependent	35		
Became independent	–	1	
Became partly dependent	–	23	
Remained utterly dependent		11	35
Partly dependent	21		
Became independent	–	6	
Remained partly dependent	–	15	21
Total	58		58

CIC, clean intermittent catheterization; IVES, intravesical electrostimulation.

**Table 3.** Pre- and Post-Intravesical Electrostimulation Urodynamic Parameters Comparison

Variables	Baseline Parameter	Post-Treatment Parameter	*P*
Qmax	7.28 ± 5.24	7.29 ± 4.09	.030*
Voided volume	161.71 ± 60.73	167.48 ± 65.63	.292
PVR	73.03 ± 43.91	62.07 ± 39.10	.005*
First sensation	115.62 ± 44.73	115.53 ± 42.36	.498
Strong sensation	223.64 ± 68.44	213.87 ± 66.06	.531
Cystometric capacity	223.73 ± 68.39	214.70 ± 65.51	.551
PDet@Qmax	17.10 ± 12.35	18.87 ± 12.47	.009*
PDetMax	20.62 ± 14.29	19.62 ± 12.47	.394

Cystometric capacity, the maximum bladder capacity; first sensation, the initial sensation of bladder filling; PDet@Qmax, detrusor pressure at the maximum flow rate; PDetMax, maximum detrusor pressure; PVR, post-void residual; strong sensation, the volume at which a person feels a strong desire to void during cystometry; Qmax, maximum flow rate; voided volume, the total volume of urine expelled.

*Wilcoxon signed-rank test; *P* < .05.
